# A novel small molecule inhibitor of CD73 triggers immune-mediated multiple myeloma cell death

**DOI:** 10.1038/s41408-024-01019-5

**Published:** 2024-04-09

**Authors:** Arghya Ray, Ting Du, Xueping Wan, Yan Song, Sindhu C. Pillai, Md. Abu Musa, Teng Fang, Jared Moore, Brian Blank, Xiaohui Du, Xi Chen, Robert Warne, Dena Sutimantanapi, Fang Lui, Tatiana Zavorotinskaya, Christophe Colas, Lori Friedman, Melissa R. Junttila, Dharminder Chauhan, Kenneth C. Anderson

**Affiliations:** 1 The LeBow Institute for Myeloma Therapeutics and Jerome Lipper Myeloma Center, Department of Medical Oncology, Dana Farber Cancer Institute, Harvard Medical School, Boston, MA USA; 2 ORIC Pharmaceuticals, Inc., South San Francisco, CA USA

**Keywords:** Cancer microenvironment, Myeloma

## Abstract

- CD73 is the key ectoenzyme involved in the generation of AMP-derived adenosine, which contributes to immunosuppression in the MM BM milieu.
- Blocking CD73 activity with a potent, selective, orally bioavailable CD73 inhibitor ORIC-533 decreases adenosine generation, overcomes immune suppression, and restores immune cell-mediated MM cell lysis.
- Based on these preclinical studies, a multi-center clinical trial of ORIC-533 has been initiated in patients with relapsed refractory MM (NCT05227144).

**Dear Editor**,

The extracellular adenosine of the tumor microenvironment is a critical immunosuppressive metabolite that impairs effector functions and enhances the proliferation of regulatory T cells and granulocytic myeloid-derived suppressor cells [1–4]. Adenosine is generated primarily by the combined effort of the ectonucleotidases CD39 and CD73 [1–4], and high CD73 expression has been observed in many cancers [4–6]. We and others have highlighted the significance of immunosuppressive adenosine signaling pathway, and the therapeutic potential of targeting CD73 in cancers including multiple myeloma (MM) [4–8]. High CD73 expression is linked to adverse prognosis in breast, lung, pancreatic, gastric, and colon cancer [1, 9–12], and adding an anti-CD73 antibody to the standard of care treatment for non-small cell lung cancer improves overall response rate and progression-free survival [12].

MM is a cancer of the plasma cells [13–16] located primarily in the bone marrow (BM), where MM cells interact with immune accessory cells to impede anti-tumor immunity and promote tumorigenesis [15–20]. Interaction between MM cells and plasmacytoid dendritic cells (pDCs) induces the transcription of CD73 in MM cells [6] correlating with an increase in adenosine production; conversely, CD73 blockade activates pDCs, and triggers T cell proliferation and decreases adenosine levels [6]. ORIC-533 (ORIC Pharmaceuticals, CA, USA), a potent, orally bioavailable, adenosine monophosphate (AMP)- competitive, highly selective small molecule inhibitor of CD73, is currently undergoing a clinical trial in MM [7, 8]. Here, we utilized our preclinical autologous coculture models of MM patient BM accessory and tumor cells to examine: (1) whether ORIC-533-mediated blockade of CD73 improves the anti-MM immunity; and (2) whether combining ORIC-533 with daratumumab, a frequently used therapeutic antibody that targets an enzyme upstream of CD73 in the adenosine generation pathway enhances anti-MM activity.

All our studies utilizing MM patient samples were performed following IRB-approved protocols at the Dana-Farber Cancer Institute and Brigham and Women’s Hospital (Boston, MA, USA). Informed consent was obtained from all patients, and patient samples were de-identified prior to their use (*in accordance with the Helsinki protocol*). Most of the bone marrow (BM) samples used here were from patients with relapsed/ refractory MM after at least three lines of therapy including proteasome inhibitors, immunomodulatory drugs, and anti-CD38 monoclonal antibodies. Autologous ex vivo assays were performed using freshly isolated BM aspirates from MM patients to investigate changes in CD138^+^ MM tumor cell viability and adenosine generation following CD73 blockade by ORIC-533. Additional information on healthy donors and MM patient BM samples, and assays utilized in the study are provided in the Supplemental Materials section.

At first, we evaluated the cellular potency of ORIC-533 using a CD73-expressing non-small cell lung cancer (NSCLC) cell line H1568 and human CD8^**+**^ T cells which efficiently convert AMP into adenosine. ORIC-533 robustly suppresses adenosine production from AMP compared to all other nucleotidase enzymes capable of adenosine conversion, as well as other closely related family member enzymes (Fig. S1B). Moreover, ORIC-533 could completely inhibit adenosine generation from AMP in H1568 cells, whereas oleclumab, a clinical stage CD73 antibody could not (Fig. 1A) [7, 8]. No direct cytotoxic effect of ORIC-533 was observed against MM cells, pDCs or normal PBMCs (Fig. S2).

**Fig. 1:**
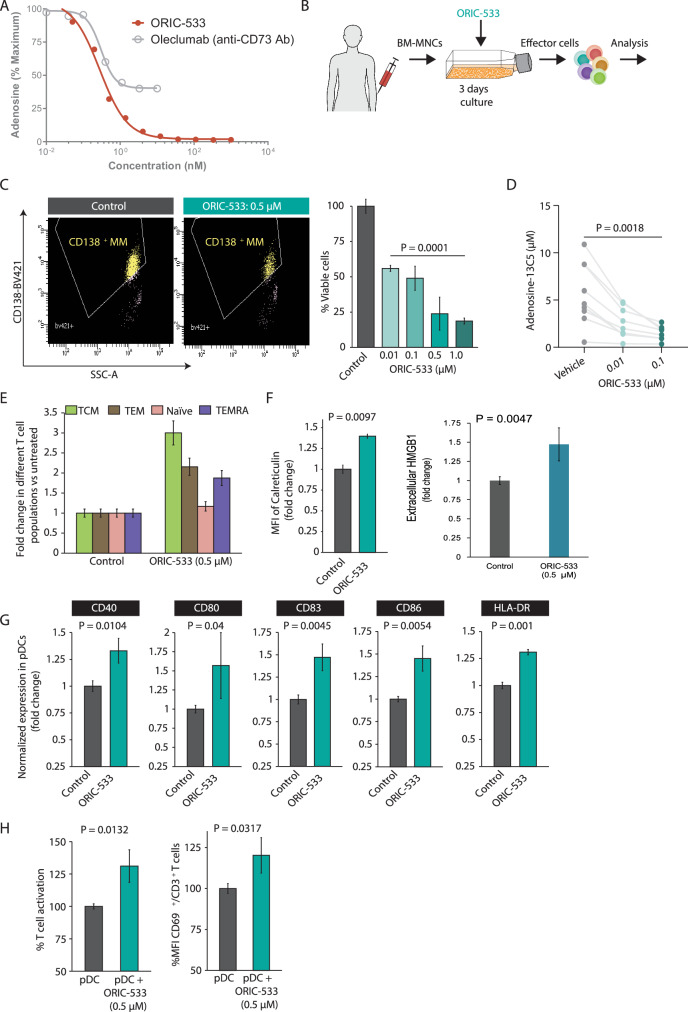
The discovery of ORIC-533 and its effect on multiple myeloma in the bone marrow microenvironment. **A** Discovery of ORIC-533: H1568 cells were pre-treated with indicated agents for 15 min, and then 10 µM AMP/5 µM EHNA was added. After 1 h, adenosine concentration in the supernatant was quantified by LC-MS/MS. **B** Experimental design of MM ex vivo assays. **C** ORIC-533 induces autologous MM cell killing in MM BM-MNCs in a dose-dependent manner: After MM patient (N = 5) total BM-MNC samples were treated with ORIC-533, they were stained with 7ADD and anti-CD138 Ab conjugated to BV421. *Left panel:* Representative FACS scatter plot of viable MM cells. *Right panel:* Quantification of FACS data (mean ± SD; p < 0.05 for all; p = 0.0001, treated with 1.0 µM of ORIC-533 *versus* untreated). **D** ORIC-533 Reduces Adenosine Production in Bone Marrow Aspirates from Relapsed/Refractory Multiple Myeloma Patients: Plasma supernatants from BM aspirates was incubated with AMP-13C5 for 15 min with or without ORIC-533. Adenosine-13C5 was quantified by MS (N = 9; P = 0.0018, treated with 0.1 µM versus untreated). **E** ORIC-533 triggers the upregulation of Central and Effector Memory T cells in MM BM-MNCs: MM patient total BM-MNCs were treated with a CD3/CD28 cocktail for 2 days to stimulate T cells. Then, cells were washed, resuspended in fresh medium, and treated with DMSO or ORIC-533 (0.5 µM) for 7–10 days. Next, cells were assessed by FACS: T-central memory (TCM: CD3^**+**^/CD62L^**+**^/CD45RA^−^), T-effector memory (TEM: CD3^**+**^/CD62L^−^/CD45RA^−^), T-terminal effector memory (TEMRA; CD3^+^/CD45RA^+^ CD62L^−^), T-naïve (Naïve: CD3^+^/CD62L^+^/CD45RA^+^) cells. **F** ORIC-533 upregulates Calreticulin expression: MM patient (N = 4) total BM-MNCs were treated with ORIC-533 (0.5 µm) or DMSO control for 3–4 days. Viable CD138^+^ MM cells were analyzed for surface expression of Calreticulin by flow cytometry (*left panel)*, and the supernatant was analyzed for HMGB1 levels (*right panel)*, using the human HMGB1/HMG-1 ELISA Kit (Novus Biologicals, CO. USA) (mean ± SD; p < 0.05). **G** ORIC-533 activates MM-BM pDCs in the MM bone marrow microenvironment: MM patient (N = 3) pDCs were treated with ORIC-533 (0.5 µM) for 24 h followed by their analysis of indicated activation/maturation markers (mean ± SD; p < 0.05). **H** ORIC-533 stimulates proliferation of CD+ T cells and enhances the activation marker CD69 on CD3+ T cells: pDCs from MM patients (N = 3) were cocultured with autologous T cells at a 1:10 (pDC:T) ratio with or without ORIC-533 (0.5 µM) for 2 days. Viable CD3^+^ T cells were analyzed for the expression of CD69 by flow (mean ± SD; P < 0.05).

We next used an ex vivo experimental model with the aim to mimic some features of the tumor microenvironment involved in clinical trials and investigated the effect of CD73 blockade with ORIC-533 in MM bone marrow mononuclear cells (BM-MNCs) (Fig. 1B). We examined whether ORIC-533 can reduce the number of viable patient autologous MM cells. We treated freshly isolated MM patient BM-MNCs (N = 5), including samples from patients with relapsed MM resistant to bortezomib, carfilzomib, dexamethasone, lenalidomide, pomalidomide and CD38 monoclonal Ab therapies, with ORIC-533 for 3–4 days, followed by quantification of viable CD138^+^ MM cells by flow cytometry. Our results showed that ORIC-533 significantly reduced the number of viable CD138^+^ cells in a dose-dependent manner (Fig. 1C; N = 5; P = 0.0001; Figure S3). To confirm that ORIC-533 blocks adenosine generation, we treated plasma supernatants of BM aspirates obtained from 9 relapsed/refractory MM patients with increasing doses of ORIC-533 and incubated them with AMP-13C5 for 15 min. Using mass spectrometry, we found that ORIC-533 inhibited adenosine-13C5 production in plasma supernatants in a dose-dependent manner (Fig. 1D; P = 0.0225 and P = 0.0018; control *versus* 0.01 µM and 0.1 µM ORIC-533, respectively).

Immunosuppression in MM is characterized by the presence of exhausted T cells, an insufficient number of central memory T cells, and high checkpoint expression on effector cells [16–20]. To investigate whether ORIC-533 can stimulate memory T cell generation in MM BM-MNCs, we analyzed the distribution of T cell subsets in response to ORIC-533 treatment of BM-MNCs (Fig. 1E; N = 4). Our results clearly show that ORIC-533 upregulated activated T cell subsets including central memory (TCM, ~3-fold, p = 0.0001), effector memory (TEM, ~2.15-fold; p = 0.001), terminal effector memory (TEMRA; ~1.87-fold; p = 0.003), and moderately naïve (p = 0.0542) T cells, indicating potential immunomodulatory properties of ORIC-533. Similar immunomodulation has also been observed for iMiDs [19, 21] and in combination therapies involving HDAC inhibitors [18, 22].

Next, we investigated whether the anti-MM immunity exerted by ORIC-533 also involves other mechanisms. Several anti-MM drugs have been reported to trigger immunogenic cell death (ICD) [23], as manifested by the upregulation of surface calreticulin and sustained release of Damage-associated molecular patterns (DAMPs) like high mobility group box 1 (HMGB 1) into the tumor-microenvironment. The pDC-MM cell interactions [6] decrease the transcription of ICD-related genes like Calreticulin (*CALR*) and *HMGB1* in MM cells (Fig. S4), but we found that ORIC-533 treatment upregulates both surface calreticulin expression on MM cells (Fig. 1F- left panel;, MFI: 1.4-fold; N = 4; P = 0.0097), and HMGB1 levels in the cell culture supernatant (N = 4; Fig. 1F- right panel) which correlates with the induction of ICD [23].

In MM, pDCs have a reduced ability to activate T cell proliferation [6, 16–18]. Importantly, ORIC-533 treatment upregulated pDC activation/maturation markers (Fig. 1G: CD40/CD80/CD83/CD86/HLA-DR; ~1.3–1.6-fold; N = 3; P < 0.05), and these activated pDCs triggered CD69 upregulation, indicating the activation of autologous T cells (Fig. 1H; N = 3, p < 0.05).

Adenosine downregulates NK cell effector function by suppressing the activity and trafficking of NK cells to the tumor site [24]. We next examined whether ORIC-533 restores NK cell-mediated anti-MM immunity. We treated MM patient total BM-MNCs with ORIC-533 for 3–4 days, the K562 cell line was added to the coculture after that, and NK cell mediated cytotoxicity was assessed from K562 lysis. K562 cells highly express ligands for NK group 2D (NKG2D) and the Natural Cytotoxicity receptors, but weakly express MHC Class-I, and are naturally susceptible to NK cell–mediated lysis. We found that ORIC-533 induced significant killing of K562 cells (Fig. 2A: N = 3; p < 0.05), without directly affecting the viability of K562 or MM NK cells (data not shown). These results indicate that ORIC-533 enhances NK-cell mediated anti-MM activity by affecting receptor-ligand (e.g., NKG2D/NKG2DL, or KIR) expression-driven tumor cell killing [17, 18, 24]. These data suggest that ORIC-533-induced anti-MM activity in MM BM-MNCs could be partly NK-cell mediated [17, 18, 24].

**Fig. 2:**
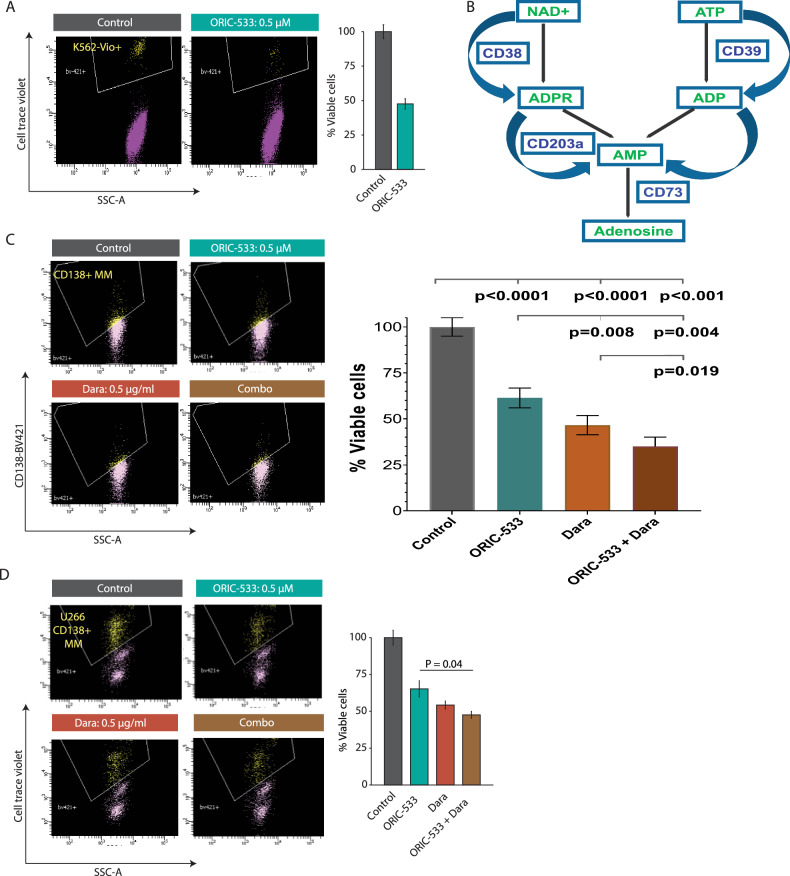
ORIC-533 induces NK cell-mediated cytotoxicity in MM and enhances autologous MM cell killing in combination with Daratumumab. **A** ORIC-533 induces MM NK cell-mediated cytotoxicity in MM BM-MNCs MM patient total BM-MNCs were treated with ORIC-533 (0.5 µM) or DMSO control for 3 days, then washed and resuspended, followed by addition of cell trace violet-stained K562 cells. Cells were incubated for 1 day before multicolor flow analysis to assess K562 cell lysis. *Left panel:* Representative FACS scatter plot of viable K562 cells. *Right panel:* Quantification of FACS data (mean ± SD; treated *versus* untreated). **B** Schematic representation of the converging pathways of ATP-dependent (CD39 > CD73) and NAD + -dependent (CD38 > CD203A > CD73) adenosine production. **C** Combination of ORIC-533 and Daratumumab enhances autologous MM cell killing: MM patient (N = 4; Supplementary Table 1) total BM-MNCs were treated with ORIC-533 (0.5 µM) or anti-CD38 Ab (Daratumumab, 0.5 µg/ml), or both for 3–4 days, and multicolor flow analysis was utilized to assess MM cell lysis. Control cells were treated with DMSO. *Left panel:* Representative FACS scatter plot of viable MM cells. *Right panel:* Quantification of FACS data (mean ± SD; p = 0.0114 for combination versus single agent). **D** Combination of ORIC-533 and Daratumumab enhances U266 MM cell killing by MM BM-MNCs MM patient (N = 4; Supplementary Table 1) total BM-MNCs were treated with ORIC-533 (0.5 µM) or anti-CD38 Ab (Daratumumab, 0.5 µg/ml), or both for 3 days. The cells were washed and pre-stained, then U266 MM cells were added to the culture, and the cells were incubated for 1 day. *Left:* Representative FACS scatter plot of viable MM cells. *Right:* Quantification of FACS data (mean ± SD; p = 0.04 for combination versus single agent).

Adenosine generation involves upstream signaling via CD39/CD73- and CD38/CD203a/CD73-axes (Fig. 2B) [1, 5, 6, 25]. Since CD38 is highly expressed in MM and the anti-CD38 antibody daratumumab is efficacious in both mono- and combination therapies in MM [26], we hypothesized that a combined blockade of the adenosine signaling pathway using both ORIC-533 and daratumumab would enhance the anti-MM immune response (Fig. 2B). Total BM-MNCs from MM patients the majority of whom (3 out 4) had prior daratumumab therapy (N = 4; Supplementary Table 1A) were treated with ORIC-533 (0.5 µM), daratumumab (0.5 µg/ml), or both agents for 3–4 days, and autologous CD138^+^ MM cell lysis was assessed using flow cytometry. Combined ORIC-533 and daratumumab triggered a more robust MM cell lysis than either agent alone (Fig. 2C; scatter plot and bar graph; p < 0.05). Importantly, the combination strategy was also effective against allogeneic HLA-A2^**+**^ U266 MM cells (Fig. S5 and Fig. 2D; N = 4; p < 0.05; Supplementary Table 1B).

Adenosine is an immunosuppressive metabolite that is generated in the bone marrow (BM) microenvironment by CD73, whose expression correlates with poor clinical outcome in many cancers including MM [6]. In the current study we utilized autologous coculture models using MM patient BM mononuclear cells (BM-MNC) and collectively our data show that: (1) CD73-blockade by ORIC-533 triggers anti-MM immunity to induce autologous tumor cell killing in MM-BM; (2) ORIC-533 triggers NK cell-mediated cytotoxicity, induces immunogenic cell death (ICD), upregulates memory T cells, activates pDCs and subsequently stimulates CD3 + /CD69 + T cells; and (3) ORIC-533 in combination with anti-CD38 antibody daratumumab enhances MM cell killing. Remarkably, ORIC-533 enhances cytolytic immune behavior both as a single agent and in combination with daratumumab in patients with prior anti-CD38 experience. Our preclinical study therefore confirms that targeting CD73 with ORIC-533 reduces adenosine generation, overcomes immune suppression, and restores MM cell lysis. These preclinical studies provided the basis for an ongoing clinical trial of ORIC-533 in relapsed and refractory MM (NCT05227144).

### Supplementary information

ArghyaRay-23-BCJ-0864R-ORIC-533-supplement-final

ArghyaRay-23-BCJ-0864R-ORIC-533-Suppl-Table-1-final

Figure S1

Figure S2

Figure S3

Figure S4

Figure S5

## Data Availability

Data not available without request and IRB review due to patient confidentiality.

## Glossary

MM: multiple myeloma
BM-MNCs: Bone marrow mononuclear cells
pDCs: plasmacytoid dendritic cells
AMP: adenosine 5’-monophosphate
