# Supplementary material for: A novel small molecule inhibitor of CD73 triggers immune-mediated multiple myeloma cell death
Source: Blood Cancer J. 2024 Apr 9;14(1):58. doi: 10.1038/s41408-024-01019-5 (PMC11004003; doi:10.1038/s41408-024-01019-5)
Supplement: Supplementary file 1 — ArghyaRay-23-BCJ-0864R-ORIC-533-supplement-final [file 41408_2024_1019_MOESM1_ESM.docx]

**Supplemental Materials Section**

**A Novel Small Molecule Inhibitor of CD73 Triggers Immune-Mediated Multiple Myeloma Cell Death**

^1^Arghya Ray^*^, Ph.D, ^1^Ting Du, Ph.D, ^1^Xueping Wan, Ph.D, ^1^Yan Song, Ph.D., ^1^Sindhu C Pillai, Ph.D., ^1^Md. Abu Musa, Ph.D., ^1^Teng Fang, M.D., Ph.D., ^2^Jared Moore, ^2^Brian Blank, ^2^Xiaohui Du, ^2^Xi Chen, ^2^Robert Warne, ^2^Dena Sutimantanapi, ^2^Fang Lui, ^2^Tatiana Zavorotinskaya, ^2^Christophe Colas, ^2^Lori Friedman, ^2^Melissa R Junttila, ^1^Dharminder Chauhan*¶, Ph.D and ^1^Kenneth C Anderson*¶, M.D.

^1^The LeBow Institute for Myeloma Therapeutics and Jerome Lipper Myeloma Center, Department of Medical Oncology, Dana Farber Cancer Institute, Harvard Medical School, Boston, MA; ^2^ORIC Pharmaceuticals, Inc. South San Francisco, CA

Running Title: Targeting CD73 with a small molecule inhibitor as immunotherapy in multiple myeloma

***¶****Joint Senior authors*

**Conflicts of Interest disclosure Conflicts of Interest disclosure**. K.C.A. is an advisor for Janssen, Pfizer and AstraZeneca; and has ownership interests in C4 Therapeutics, Dynamic Cell Therapies, Window Therapeutics, Starton Therapeutics, NextRNA and OncoPep. DC is consultant to Stemline Therapeutic, Inc., and an equity owner in C4 Therapeutics. AR, TD, XW, YS, SCP, MAM, and TF have no competing financial interests. J.M., B.B., X.D., C.C., R.W., D.S., T.Z., L.F. and M.R.J. are employees and equity owners of ORIC Pharmaceuticals, Inc.

*** Correspondence** Dharminder Chauhan, Ph.D ([Dharminder_Chauhan@dfci.harvard.edu](mailto:Dharminder_Chauhan@dfci.harvard.edu)); Kenneth C Anderson, M.D. [Kenneth_Anderson@dfci.harvard.edu](mailto:Kenneth_Anderson@dfci.harvard.edu)); Arghya Ray, Ph.D (arghya_ray@dfci.harvard.edu); Dana-Farber Cancer Institute, SM608, 450 Brookline Ave, Boston, MA; AR: Ph#: 617-632-2332; DC: Ph#: 617-632-4563; KCA: Ph#: 617-632-2144; Fax#: 617.632.2140

**Keywords:** Multiple Myeloma, CD73, Immunotherapy, Plasmacytoid Dendritic Cells, Adenosine, ORIC-533

**Text count**: 1497 words. 2 main Figures, 5 Supplementary Figures, and 1 Supplementary Table.

**Category: *Correspondence***

**Materials and Methods**

***CD73 Biochemical Assay*** Activity of human recombinant CD73 (R&D Systems, cat# 5797EN) was measured by quantification of free phosphate using Malachite Green Phosphate Detection Kit (R&D Systems, cat# DY996). Stock solution of ORIC-533 was dispensed into 384-well polystyrene plates using Echo 555 acoustic dispenser (Labcyte Inc.) in a 10-point half-log titration in duplicates. Then 25 µL of 0.64 nM CD73 enzyme in assay buffer (25 mM Tris-HCl, pH 7.5, 0.01% Brij-35, 0.01% BSA, 5 mM MgCl2) was added to the plates. The ORIC-533 compound and CD73 enzyme were incubated for 15 minutes at RT, followed by addition of 25 µL of 30 µM AMP (Sigma, cat# A1752), then the reaction mixture was further incubated for 10 minutes at RT. The final concentration of CD73 and AMP in the reaction were 320 pM and 15 µM, respectively. The reaction was stopped by adding 10 µL of Malachite Green reagent A and incubating for 10 minutes at RT. This was followed by addition of 10 µL Malachite Green reagent B with incubation for 45 minutes at RT. Absorbance was read at 620 nm on an EnVision plate reader (PerkinElmer). The activities of recombinant CD39/ENTPD1, CD39L3/ENTPD3 and CD39L4/ENTPD5 were measured by quantification of free phosphate generated from AMP using the Malachite Green detection system (R&D Systems, catalog #DY996). The activities of recombinant ENPP1 and ENPP7 were measured by quantification of p-nitrophenolate generated from a substrate, p-Nph-5’-TMP. Activity of recombinant SAHH was measured by quantification of free homocysteine using the SAHH Inhibitor Screening detection system (Abcam, catalog #ab204694). The activities of recombinant phosphodiesterases were measured by quantification of AMP generated from cAMP, using the Transcreener AMP/GMP FP detection system (BellBrook Labs, catalog #3015). The activities of recombinant ACPP, TNAP, NT5C2, NT5C3B and NT5M were measured by quantification of the reaction product 4-MU generated from a substrate, 4-MUP, following incubation with the enzymes.

**Purification of MM patient BM pDCs, T cells, NK cells and CD138^+^ tumor cells** Studies with patient MM cells were performed following IRB-approved protocols at Dana-Farber Cancer Institute and Brigham and Women’s Hospital (Boston, MA, USA). Informed consent was obtained from all patients, and patient samples were de-identified prior to their use (*everything in accordance with the Helsinki protocol*). pDCs were isolated from BM by magnetically activated cell sorting using CD304 (BDCA-4/Neuropilin-1) microbeads kit (Miltenyi Biotec, Auburn, CA, USA), as previously described [1-6]. The purity of pDCs (CD3-, CD14-, CD19-, CD20-, CD56-, CD11c-, MHC-II/CD123/BDCA-2**^+^**) was confirmed, as previously described [1, 3, 5]. Raw data was analyzed using FACS Diva (BD Biosciences, USA) and FlowJo (Tree Star Inc, USA). MM patient cells were purified (>95% purity) by positive selection using CD138 Microbeads kit. CD8**^+^** T cells and CD56+ NK cells were purified using negative selection immunomagnetic separation techniques, as previously described [4-7].

***Cell culture and reagents*** MM cells were cultured in complete RPMI 1640 medium, and MM cell viability was determined using WST-1 cell proliferation reagent. WST-1 Cell Proliferation Reagent was purchased from Clontech Laboratories, Inc. (USA). MM-pDCs were cocultured either in DCP-MM medium (Mattek Corp. Ashland, MA) or complete RPMI-1640 medium supplemented with IL-3 (Peprotech Inc., Rocky Hill, NJ, USA). CD3-PE/FITC/APC; CD4-FITC/PE or APC-Cy7; CD8-APC/FITC, CD56-PE; CD123-PE/PE-Cy5/FITC; and CD138-FITC/PE/APC were obtained from BD Biosciences (San Jose, CA). BDCA-2-FITC and CD11c-APC were obtained from Miltenyi Biotec (Auburn, CA); CD303-, CD304-, CD107a, CD138-BV421, Calreticulin-APC, and CD123-PE were purchased from Biolegend (USA). ImmunoCult™ Human CD3/CD28 T Cell Activator was obtained from StemCell Technologies (Cambridge MA). Immunomagnetic separation kits were purchased from Miltenyi Biotec. The CellTrace Violet and CellTracker Violet/Green flow assay kits were obtained from Life Technologies (USA). ORIC-533 was developed at ORIC Pharma (CA, USA). Daratumumab was obtained from Selleck Chemicals (USA).

***RNA sequencing using next generation sequencing*** Purified MM patient pDCs were cocultured with autologous MM cells or allogeneic MM cell lines (1pDC:5MM) for 48h, followed by separation of MM cells from pDCs using flow cytometry. Total RNA from MM cells was subjected to RNAseq analysis using Illumina Next Generation Sequencing (NGS). Raw sequence data were analyzed using VIPER workflow generating differential expression (DEseq2) and KEGG pathway [8]. The Linear model for RNAseq analysis (Limma) and its GUI (Glimma) were used for the visualization of data. Statistical significance: log2FC (fold change) values in coculture vs control, with an FDR (False Discovery Rate) value of <0.05, was considered significant (CI > 95). Pathway analysis was done using PATHVIEW (https://pathview.uncc.edu/) or pathview R package [9]. The heatmap analysis was also performed using Morpheus software (Broad Institute, MIT). Gene expression studies were validated at the protein levels using multicolor flow cytometry.

***Adenosine assay by Mass Spectrometry***  Plasma supernatants from BM aspirates from relapsed refractory MM patients was incubated with AMP-13C5 for 15 minutes in presence or absence of ORIC-533. Two doses of ORIC-533 were assessed.  Thereafter, the adenosine-13C5 was quantified by mass spectrometry as previously described (ref: https://pubs.acs.org/doi/10.1021/acs.jmedchem.0c01086). Of note, most of the BM samples used here were from patients with relapsed/ refractory MM after at least three lines of therapy including proteasome inhibitors (bortezomib, carfilzomib), immunomodulatory drugs (lenalidomide, pomalidomide and dexamethasone), and anti-CD38 monoclonal antibodies (Daratumumab).

***Cell viability assays*** MM cell lines, pDCs from MM BM samples, and PBMCs from normal healthy donors were treated with increasing concentrations of CD73 inhibitor ORIC-533 for 72h (or 48h for PBMCs) followed by the assessment of viability using WST assay, as previously described [1-7].

***Cytotoxicity Assay with BM-MNCs***: MM patient total BM-MNCs were treated with different concentrations of ORIC-533 (or with daratumumab for combination studies) for 3-4 days, and multicolor flow analysis was utilized to assess MM cell lysis. Autologous CD138^+^ MM cells were selected based on their staining of CD138-FITC Ab and quantified.

For ORIC-533-triggered (± combination) allogeneic cell U266 MM cell killing by MM BM-MNCs, MM patient total BM-MNCs were treated with ORIC-533 (0.5 µM) or anti-CD38 Ab (Daratumumab, 0.5 µg/ml), or both for 3 days. After 3 days, the cells were washed and pre-stained (Cell Trace Violet, Molecular Probes, Fisher Scientific) U266 MM cells were added to the culture, and the cells were incubated for 1 day. Following this, a multicolor flow analysis was utilized to assess U266 cell lysis. Control cells were treated with DMSO. Cells were stained with 7ADD and subjected to flow analysis. 7ADD negative cells were gated out and viable Cell Trace positive U266 cells were quantified. The fold change in viability after treatment was obtained after normalization with control data and presented as percentage of viable cells in the presence *versus* absence of different treatments.

To assess the ORIC-533-mediated NK cell activation and NK cell mediated cytotoxicity, MM patient total BM-MNCs were treated with increasing concentrations of ORIC-533 for 3 days. Control cells were treated with DMSO. After 3 days, the cells were washed and pre-stained (Cell Trace Violet, Molecular Probes, Fisher Scientific) K562 cells were added to the culture, and the cells were incubated for 1 day. K562 expressed a higher intensity of ligands for Natural Killer G2D and the Natural Cytotoxicity Receptors, and weakly express MHC Class-I. These cells are used in multicolor flow analysis of NK-cell mediated lysis. After incubation for 1 day, these cells were subjected to a multicolor flow to assess K562 cell lysis. Cells were stained with 7ADD and subjected to flow analysis. 7ADD negative cells were gated out and viable Cell Trace positive K562 cells were quantified. The fold change was obtained after normalization with control data and presented as percentage of viable cells in the presence versus absence of different treatments.

***Generation of Central and Effector Memory T cells in MM BM-MNCs:*** MM patient BM-MNCs cells were treated with CD3/CD28 cocktail for 2 days to stimulate T cells. After 2 days, cells were washed, resuspended in fresh medium, and treated with control DMSO or ORIC-533 (0.5 µM) for 7-10 days; medium changed after every 3 days. This was followed by quantification of CD3^+^T cell populations positive for CD62L+ and/or CD45RA+ using flow cytometry (N=4 MM patient BM). The cells were first gated on the basis of their CD3 expression (to identify the CD3**^+^** populations), followed by selection of CD62L^+^ and/or CD45RA^+^ single or double-positive populations, and subsequent quantification of T-central memory (TCM: CD3**^+^**/CD62L**^+^**/CD45RA**^-^** ), T-effector memory (TEM: CD3**^+^**/CD62L**^-^**/CD45RA**^-^** ), T-terminal effector memory (TEMRA ; CD3^+^/CD45RA ^+^ CD62L^−^ ), T-naïve (Naïve: CD3+/CD62L+/CD45RA +) cells [10-12].

***Characterization of Immunogenic Cell Death (ICD):*** The immunogenic cell death triggered by ORIC-533 was characterized as follow: *Flow cytometric analysis of the surface expression of Calreticulin:* MM patient (N=4) total BM-MNCs were treated with CD73 inhibitor ORIC-533 (0.5 µM) or DMSO control for 3-4 days, and multicolor flow analysis was utilized to assess MM cell lysis. Simultaneously, the cells were also stained with anti-Calreticulin Ab conjugated to a chromophore. 7AAD negative cells were gated out and from that population. Viable CD138^+^ MM cells were analyzed for the surface expression of Calreticulin to indicate ICD [13, 14].

***High Mobility Group Box 1 (HMGB1) Elisa Assay***: HMGB1 is one of the DAMPs (Damage-associated Molecular Patterns) that is released by the dying cells and is one of the important makers of ICD [13, 14]. MM patient (N=4) total BM-MNCs were treated with different concentrations of ORIC-533 or DMSO control for 3-4 days, after which cell culture supernatants were collected. The supernatants were analyzed for HMGB1 using human HMGB1/HMG-1 ELISA Kit (Novus Biologicals, CO. USA) following manufacturer’s instructions. The data is quantified as fold change in the amount or extracellular HMGB1 in the presence *versus* absence of ORIC-533.

Statistical Analysis Statistical significance was obtained using Student’s *t test,* with the minimal level of significance at p value < 0.05 (Graph Pad PRISM version 8).

**References**

1. Chauhan D, Singh AV, Brahmandam M, Carrasco R, Bandi M, Hideshima T, *et al*. Functional interaction of plasmacytoid dendritic cells with multiple myeloma cells: a therapeutic target. *Cancer Cell*. 2009;**16**: 309-323.
2. Ray A, Tian Z, Das DS, Coffman RL, Richardson P, Chauhan D *et al*. A novel TLR-9 agonist C792 inhibits plasmacytoid dendritic cell-induced myeloma cell growth and enhance cytotoxicity of bortezomib. *Leukemia.* 2014; **28**: 1716-1724.
3. Ray A, Das DS, Song Y, Richardson P, Munshi NC, Chauhan D *et al*. Targeting PD1-PDL1 immune checkpoint in plasmacytoid dendritic cell interactions with T cells, natural killer cells and multiple myeloma cells. *Leukemia*. 2015; **29**:1441-1444.
4. Ray A, Das DS, Song Y, Hideshima T, Tai YT, Chauhan D *et al.*  Combination of a novel HDAC6 inhibitor ACY-241 and anti-PD-L1 antibody enhances anti-tumor immunity and cytotoxicity in multiple myeloma. *Leukemia*. 2018; **32**: 843-846.
5. Ray A, Song Y, Du T, Tai YT, Chauhan D, Anderson KC. Targeting tryptophan catabolic kynurenine pathway enhances antitumor immunity and cytotoxicity in multiple myeloma. *Leukemia.* 2020; **34**: 567-577.
6. Ray A, Das DS, Song Y, Macri V, Richardson P, Brooks CL *et al.* A novel agent SL-401 induces anti-myeloma activity by targeting plasmacytoid dendritic cells, osteoclastogenesis and cancer stem-like cells. *Leukemia*. 2017; **31**: 2652-2660.
7. Ray A, Song Y, Du T, Chauhan D, Anderson KC. Preclinical validation of Alpha-Enolase (ENO1) as a novel immunometabolic target in multiple myeloma. *Oncogene*. 2020; **39**: 2786-2796.
8. Cornwell M, Vangala M, Taing L, Herbert Z, Köster J, Li B *et al.* VIPER: Visualization Pipeline for RNA-seq, a Snakemake workflow for efficient and complete RNA-seq analysis. *BMC Bioinformatics*. 2018; **19**: 135.
9. Luo W, Brouwer, C (2013). “Pathview: an R/Bioconductor package for pathway-based data integration and visualization.” *Bioinformatics*. 2013; **29:** 1830-1831.
10. Zelle-Rieser C, Thangavadivel S, Biedermann R, Brunner A, Stoitzner P, Willenbacher E *et al.*  T cells in multiple myeloma display features of exhaustion and senescence at the tumor site. *J Hematol Oncol*. 2016; **9**, 116.
11. Busch A, Zeh D, Janzen V, Mügge LO, Wolf D, Fingerhut L, *et al.* Treatment with lenalidomide induces immunoactivating and counter-regulatory immunosuppressive changes in myeloma patients. *Clin Exp Immunol*. 2014; **177**: 439-453.
12. Burke B, Eden C, Perez C, Belshoff A, Hart S, Plaza-Rojas L, *et al.* Inhibition of Histone Deacetylase (HDAC) Enhances Checkpoint Blockade Efficacy by Rendering Bladder Cancer Cells Visible for T Cell-Mediated Destruction. *Front Oncol*. 2020; **10**: 699.
13. Gulla A, Morelli E, Samur MK, Botta C, Hideshima T, Bianchi G, *et al.* Bortezomib induces anti-multiple myeloma immune response mediated by cGAS/STING pathway activation. *Blood Cancer Discov*. 2021; **2**: 468-483.
14. Fucikova J, Kepp O, Kasikova L, Petroni G, Yamazaki T, Liu P, *et al.* Detection of immunogenic cell death and its relevance for cancer therapy. *Cell Death Dis.* 2020; **11**, 1013
15. Ray A, Song Y, Du T, Buon L, Tai YT, Chauhan D, *et al.*  Identification and validation of ecto-5' nucleotidase as an immunotherapeutic target in multiple myeloma. *Blood Cancer J*. 2022; **12**: 50.

**Figure Legends**

**Figure S1 A)** ORIC-533 has picomolar potency in biochemical assay and effectively inhibits in vitro adenosine generation from AMP in H1568 cells at sub nanomolar concentrations, as quantified by LC-MS/MS.

**B)** ORIC-533 has an IC_50_ of 89 pM ± 20 pM (N=12) against CD73 as measured by malachite green detection system. Biochemical selectivity profile of ORIC-533 against 19 representative enzymes encompassing families of 5’-nucleotidases, ectonucleotidases, ecto-nucleotide pyrophosphatases/phosphodiesterases, phosphodiesterases and an adenosyl homocysteinase. Average IC_50_ displayed (N=2)

**Figures S2**: **Effect of CD73 inhibitor ORIC-533 on the viability of MM cell lines: (A)** MM patient BM pDCs, myeloma cell lines (**B**) MM.1S and (**C**) AMO-1, and (**D**) PBMCs from normal healthy donors were treated with increasing concentrations of CD73 inhibitor ORIC-533 for 72h (A-C) or 48h (D), followed by the assessment of viability. The bar plots indicate change in %viability versus different concentrations of the inhibitor. (mean ± SD; p < 0.05; N = 2 normal PBMC samples tested)

**Figure S3: Blockade of CD73 by ORIC-533 induces autologous MM cell killing in MM BM-MNCs:** As in Figure 1C, MM patient (N=3) total BM-MNCs were treated with CD73 inhibitor ORIC-533 (1.0 µm) or DMSO control for 3-4 days, and multicolor flow analysis was utilized to assess MM cell lysis. Cells were stained with 7ADD and anti-CD138 Ab conjugated to BV421. 7ADD negative cells were gated out and from that population, and CD138^+^ MM cells were quantified. ***Left panel****:* Representative FACS scatter plot showing a decrease in number of viable BV421+ autologous MM cells after the treatment with CD73 inhibitor ORIC-533. ***Right Panel:*** Bar graph shows quantification of CD138^+^ MM cells in left panel. The fold change was obtained after normalization with control data, and is presented as percentage of viable cells in the presence versus absence of CD73 inhibitor ORIC-533 (mean ± SD; p < 0.05).

**Figure S4: Effects of pDC-MM interaction on the transcription of ICD-related genes in MM:** Purified MM patient pDCs (from 3 MM patients) were co-cultured with MM.1S cells (1pDC/5MM) for 48h, followed by separation of MM.1S cells from pDCs using flow cytometry. Total RNA from MM.1S cells was subjected to RNAseq analysis [15]. RNA-seq analysis showed downregulation of **(A)** *CALR* (Calreticulin) *and (****B)*** *HMGB1* (High mobility group box 1) genes in MM after pDC-MM co-culture *versus* MM cells cultured alone [Calreticulin: 0.6833-fold *versus* MM alone, Adjusted p = 3.49E-18; HMGB1: 0.375581281 –fold *versus* MM alone, Adjusted p = 9.44E-34; N = 3; MM: Myeloma cells cultured alone, pDC-MM: MM cells cultured with pDC and then flow separated after the coculture].

**Figure S5:** Schematic representation of the cytotoxic assay using U266 MM cells.

**Supplementary Table 1A-B: Information about the prior therapies of MM patients.**

**Supplementary Table 1A:** Prior therapies of patients whose bone marrow samples were used for the study presented in Figure 2C**.**

**Supplementary Table 1B:** Prior therapies of patients whose bone marrow samples were used for the study presented in Figure 2D.
