# Supplementary material for: A novel small molecule inhibitor of CD73 triggers immune-mediated multiple myeloma cell death
Source: Blood Cancer J. 2024 Apr 9;14(1):58. doi: 10.1038/s41408-024-01019-5 (PMC11004003; doi:10.1038/s41408-024-01019-5)
Supplement: Supplementary file 2 — ArghyaRay-23-BCJ-0864R-ORIC-533-Suppl-Table-1-final [file 41408_2024_1019_MOESM2_ESM.docx]

**Supplementary Table 1A: Prior therapies of patients whose bone marrow samples were used for the study presented in Figure 2C.**

| **Patient No** | **Treatments** | | | | | | |
| --- | --- | --- | --- | --- | --- | --- | --- |
|  | **1** | **2** | **3** | **4** | **5** | **6** | **7** |
| **1** | **Rev/Vel/ Dex** | **Ixazomide Pom/ Dex** |  |  |  |  |  |
| **2** | **RVD** | **CyBorD** | **Dox/Vel/ ThD/ Dex** | **ASCT** | **Vel/ Dex/ Pom** | **Elo/ Pom/ Dex** | **Dara/ CC92480/ Dex** |
| **3** | **Dox KRD** | **KR or KRD** | **Isa/ Pom/ Dex** | **Next on to DCEP** |  |  |  |
| **4** | **RVD** | **Pom Dex** | **Ixa/ Pom/ Dex** | **Dara/ Carfi /Pom/ Dex** |  |  |  |
|  |  |  |  |  |  |  |  |

**Supplementary Table 1B: Prior therapies of patients whose bone marrow samples were used for the study presented in Figure 2D.**

| **Patient No** | **Treatments** | | | | | | | | | | |
| --- | --- | --- | --- | --- | --- | --- | --- | --- | --- | --- | --- |
|  | **1** | | **2** | **3** | | **4** | | **5** | **6** | **7** | **8** |
| **1** | **RVD** |  | | |  |  |  | |  |  |  |
| **2** | **RVD** | **DCEP** | | | **CyBorD** |  |  | |  |  |  |
| 3 | **RVD** | **RVD elo** | | | **Pom Dex** | **Carfi Dara Dex** | **Belantamab** | | **Melflufen/ Vel/ Dex/** | **CC92480/ Dex** |  |
| 4 | **RVD** | **ASCT** | | | **Ixa/ Dex** | **CyBorD** | **Carfi/ Dex** | | **Carfi/ Dex/ Dara** | **Pom/ Bort/ Dex** | **Elo/ Pom/ Dex** |
|  |  |  | | |  |  |  | |  |  |  |

**Glossary:**

**ASCT: Autologous Stem Cell Transplantation**

**Belantamab**: Belantamab mafodotin

**Carf Dara Dex**: Carfilozomib, Daratumumab and Dexamethosone

**CC92480:** CC-92480 Is a Novel Cereblon E3 Ligase Modulator (CELMoD)

**Dara/ Carfi /Pom/ Dex**: Daratumumab, Carfilizomob, Pomalidomide and

Dexamethasone

**DCEP**: Dexamethasone, Cyclophosphamide, Etoposide, and

Cisplatin.

**Dox/Vel/ Thd/ Dex**: Doxorubicin, Velcade, Thalidomide and Dexamethasone

**Elo/Pom/Dex**: Elotuzumab, Pomalidomide and Dexamethasone.

**Isa/Pom/Dex**: Isatuximab (anti-C38 Ab), Pomalidomide and

Dexamethasone.

**Ixa/Pom/Dex**: Ixazomib (Ninlaro), Pomalidomide and Dexamethasone

**KR:** Carfilozomib and Lenalidomide.

**KRD (KRd):** Carfilozomib, Lenalidomide, Dexamethosone

**Pom/Bort/Dex**: Pomalidomide, Bortezomib and Dexamethasone.

**RVD:** Revlimid, Velcade and Dexamethasone.

**RVD elo:** RVD plus Elotuzumab (Empliciti)
