# Supplementary figures and images for: A novel small molecule inhibitor of CD73 triggers immune-mediated multiple myeloma cell death
Source: Blood Cancer J. 2024 Apr 9;14(1):58. doi: 10.1038/s41408-024-01019-5 (PMC11004003; doi:10.1038/s41408-024-01019-5)

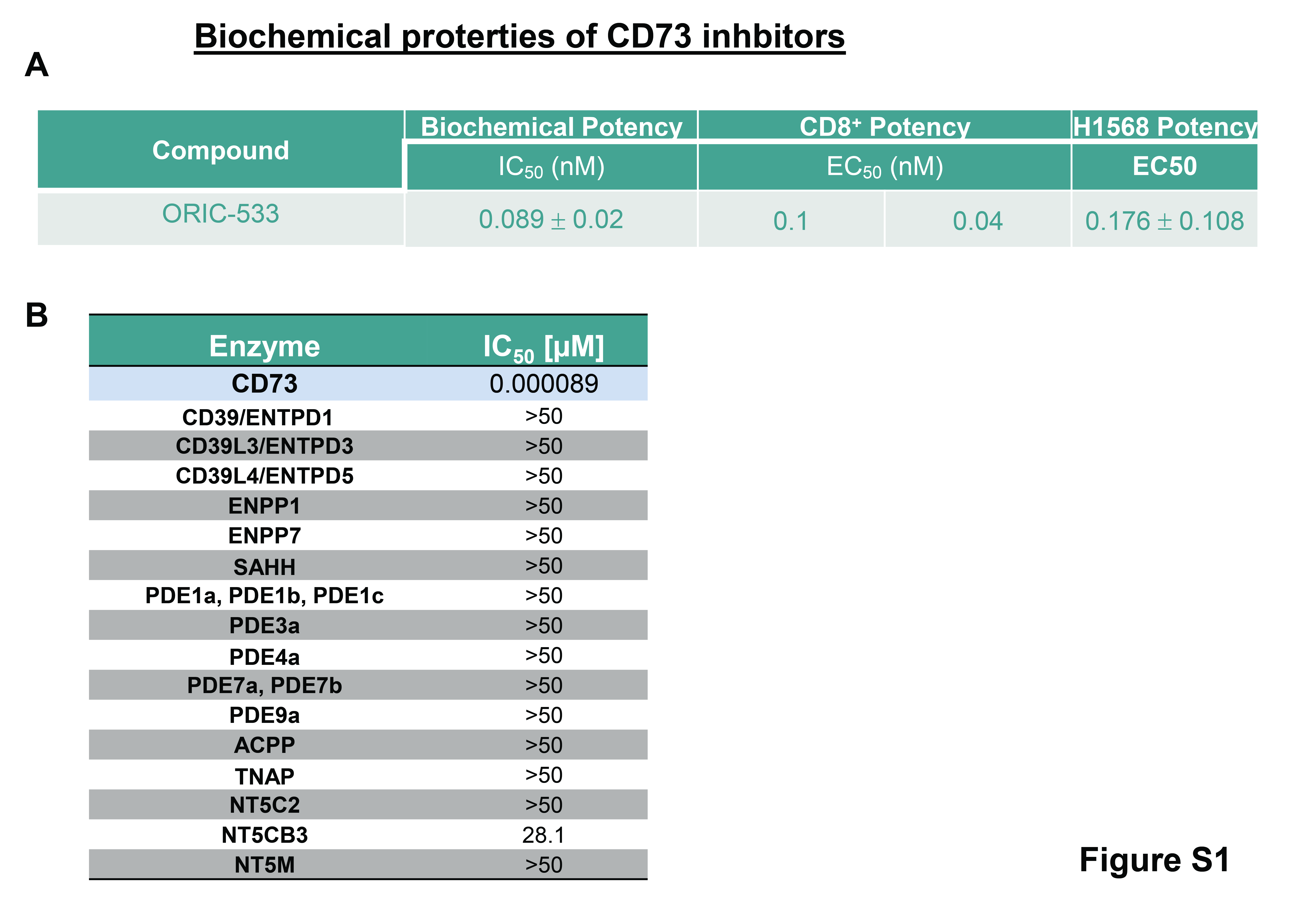

Supplement: Supplementary file 3 — Figure S1 [file 41408_2024_1019_MOESM3_ESM.tif]

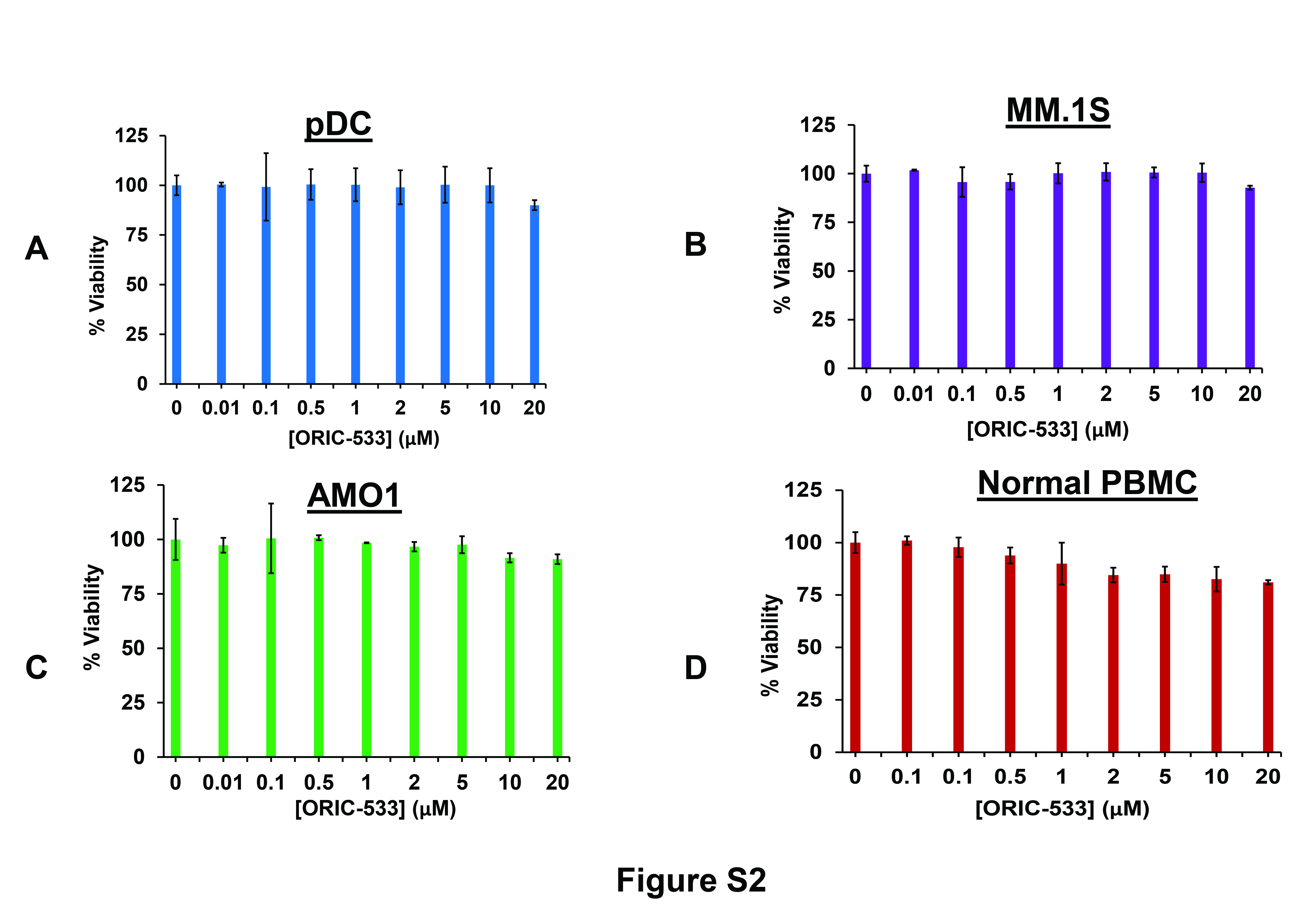

Supplement: Supplementary file 4 — Figure S2 [file 41408_2024_1019_MOESM4_ESM.tif]

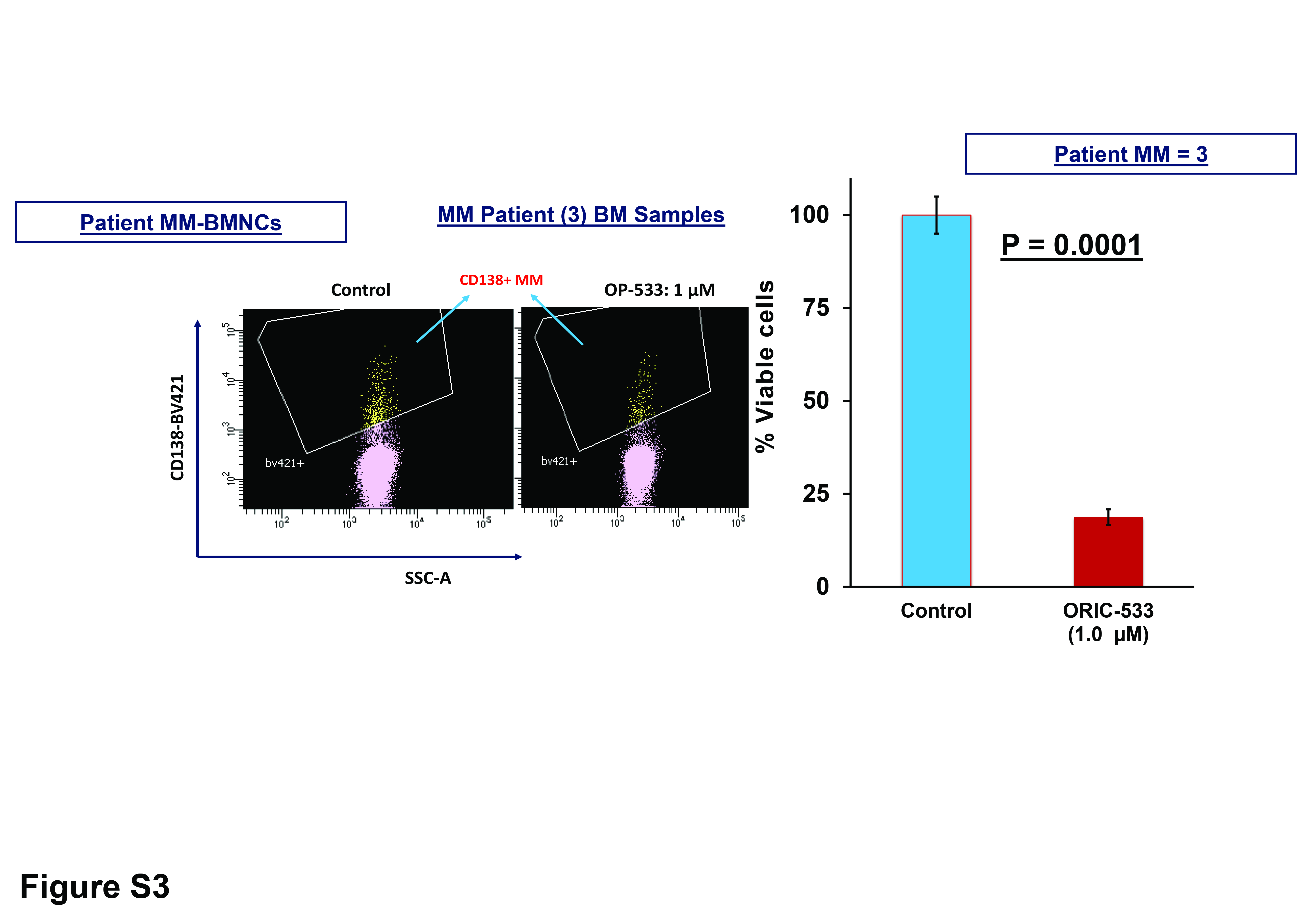

Supplement: Supplementary file 5 — Figure S3 [file 41408_2024_1019_MOESM5_ESM.tif]

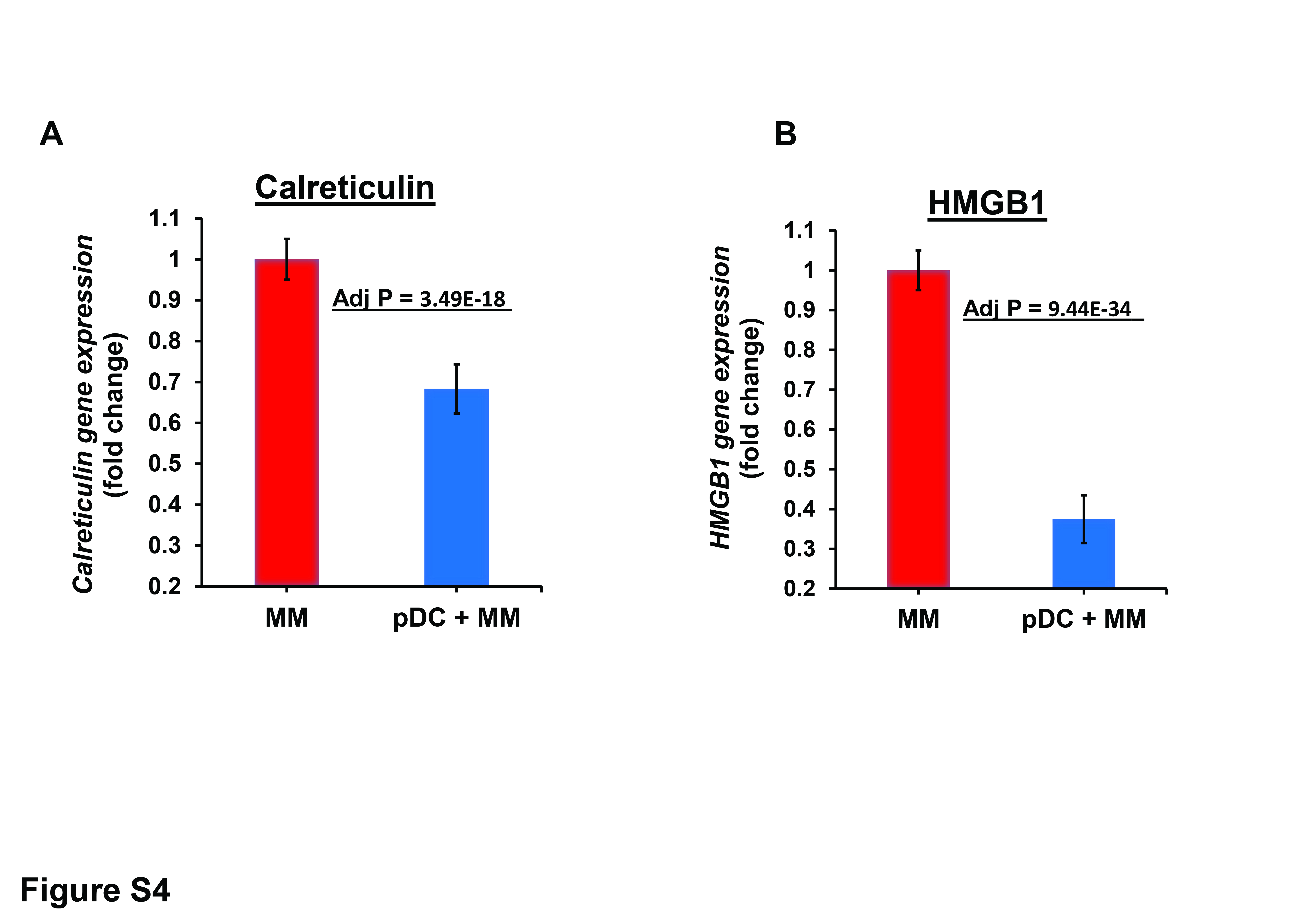

Supplement: Supplementary file 6 — Figure S4 [file 41408_2024_1019_MOESM6_ESM.tif]

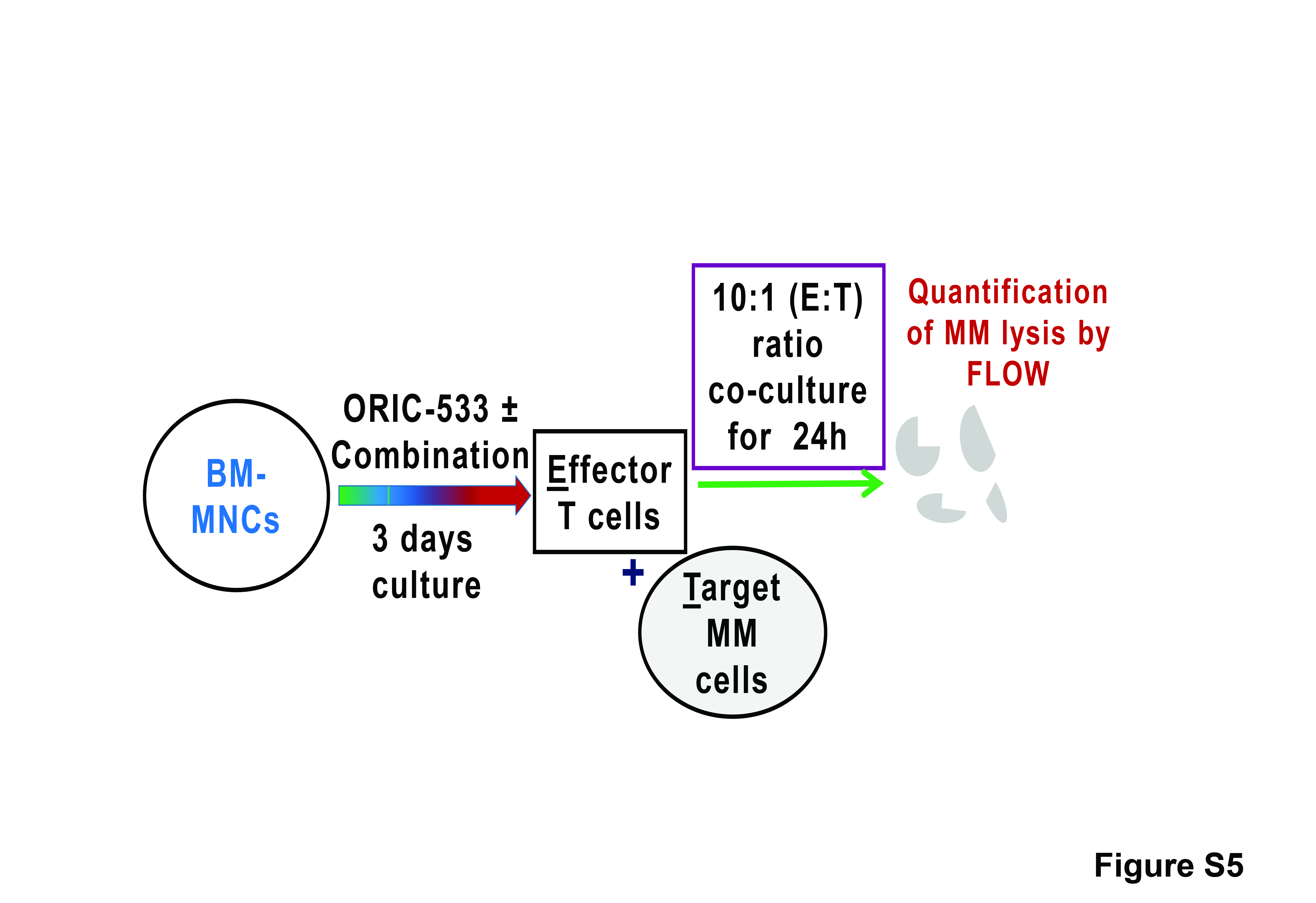

Supplement: Supplementary file 7 — Figure S5 [file 41408_2024_1019_MOESM7_ESM.tif]
